# Evaluating The Therapeutic Effect of 2-Nitroimidazole on *Toxoplasma gondii*: An In vitro and In vivo Study Using BALB/c Mice

**DOI:** 10.5812/ijpr-157086

**Published:** 2025-02-24

**Authors:** Elaheh Ghiasipour, Javid Sadraei, Fatemeh Ghaffarifar

**Affiliations:** 1 Department of Parasitology, Faculty of Medical Sciences, Tarbiat Modares University, Tehran, Iran

**Keywords:** *Toxoplasma gondii*, 2-Nitroimidazole, Sulfadiazine, Tachyzoite, In vitro, In vivo

## Abstract

**Background:**

Toxoplasmosis, caused by the protozoan parasite *Toxoplasma gondii*, remains a significant health concern due to its widespread prevalence and severe impact on immunocompromised individuals. Current treatments are limited, necessitating the exploration of new therapeutic agents.

**Objectives:**

This study aimed to evaluate the efficacy and safety of 2-nitroimidazole as a potential treatment for toxoplasmosis in BALB/c mice, comparing its effects with the standard treatment, sulfadiazine.

**Methods:**

In vitro assays were conducted to determine the half-maximal inhibitory concentration (IC50) of 2-nitroimidazole and sulfadiazine against *T. gondii* tachyzoites. The MTT assay was used to assess the cytotoxicity of 2-nitroimidazole on macrophages. In vivo experiments involved treating BALB/c mice infected with *T. gondii* with either 2-nitroimidazole or sulfadiazine, monitoring survival rates and therapeutic outcomes.

**Results:**

In vitro results revealed IC50 values of 5.43 μM for 2-nitroimidazole and 2.99 μM for sulfadiazine, indicating potent anti-tachyzoite activity. The MTT assay showed that 2-nitroimidazole had low cytotoxicity, with significant cell viability even at higher concentrations. Based on the MTT assay findings, 40 μM of 2-nitroimidazole showed the highest level of toxicity towards macrophages. Furthermore, flow cytometry analysis revealed that this compound induced apoptosis in approximately 58.9% of tachyzoites. In vivo, all mice in the control group died by the eighth day. Treatment with sulfadiazine resulted in two mice surviving until the 14th day, while 2-nitroimidazole treatment saw one mouse surviving to the same day. These findings suggest that 2-nitroimidazole has comparable efficacy to sulfadiazine with potentially fewer side effects.

**Conclusions:**

The study demonstrates that 2-nitroimidazole is a promising candidate for the treatment of toxoplasmosis, exhibiting strong anti-parasitic activity and low cytotoxicity. Further research is warranted to optimize dosing regimens and explore combination therapies to enhance its therapeutic potential.

## 1. Background

*Toxoplasma gondii*, a ubiquitous and obligatory intracellular protozoan parasite of the Apicomplexa phylum, infects a wide range of vertebrates, including humans, causing the neglected disease toxoplasmosis (1). Cats are the definitive hosts of this parasite throughout its life cycle, and by excreting oocysts, they can infect any warm-blooded creatures, including humans, birds, and mammals, as well as the environment, food, and water (2, 3). Inadequate hygiene practices are a critical factor in the transmission of toxoplasmosis, and studies show that about 30% of the world's population is infected with *T. gondii* (4).

In immunocompetent hosts, toxoplasmosis typically manifests as an asymptomatic infection or presents with mild clinical signs, such as lymphadenopathy and influenza-like symptoms. However, the primary concern regarding toxoplasmosis is its impact on immunocompromised individuals, including those with malignancies, HIV/AIDS patients, and organ transplant recipients. In these susceptible populations, the disease exhibits a broad spectrum of symptomatology with varying severities, encompassing encephalitis, seizures, vision disorders, and poor coordination. If left untreated, these manifestations can escalate, potentially leading to fatal outcomes (5, 6).

Pyrimethamine and sulfadiazine are the first-line medications used to treat and prevent toxoplasmosis. These medications cause a variety of adverse effects, including neutropenia, leukopenia, thrombocytopenia, a significant decline in platelet count, blood abnormalities, allergic reactions, and increased serum creatinine and serum liver enzymes (7). Other chemical agents used to treat toxoplasmosis show limited efficacy. Like other parasites, cases of drug resistance have been reported for *T. gondii* (8).

Prevention is primarily recommended for seronegative pregnant women and immunocompromised patients with CD4 counts below 100 cells/μL as the ideal strategy to prevent this disease. However, despite extensive research on vaccine development, no acceptable and successful vaccine for toxoplasmosis has been established (9-11). Therefore, we require medications that are less toxic and more effective against the parasite at all phases of its life cycle, including bradyzoites in tissues (8).

In recent years, research on the effectiveness of herbal products and alternative medicines for various diseases, including parasitic diseases, has increased (12). Laboratory and experimental studies have demonstrated that certain plant extracts and fractions exhibit significant anti-parasitic effects on diseases caused by protozoan and helminthic parasites such as *Leishmania*, *Trypanosoma*, *Toxoplasma*, and *Echinococcus* *species* (13, 14).

Despite the availability of standard treatments like pyrimethamine and sulfadiazine, their associated clinical side effects have prompted researchers to investigate alternative solutions. For instance, studies on chitosan nanoparticles have highlighted their potential as antiparasitic agents, with low molecular weight formulations demonstrating superior efficacy against *T. gondii* tachyzoites in both in vitro and in vivo settings (15). Additionally, green-synthesized copper nanoparticles, particularly in combination with atovaquone, have shown promising results in models of chronic toxoplasmosis by significantly reducing tissue cysts and boosting immune responses (16). These advancements emphasize the promise of nanotechnology-based therapies in addressing the limitations of traditional treatments while offering safer and more effective options.

Nitroimidazoles are a group of nitroheterocyclic compounds with broad-spectrum activity against parasites, mycobacteria, and gram-positive and gram-negative bacteria (17, 18). The ability of pharmaceutical compounds like nitroimidazoles, nitrothiazoles, and nitrofurans to effectively combat infectious organisms is highly dependent on the presence of nitro groups, notwithstanding the possibility of toxicity (19). In the early 1950s, the discovery of azomycin, a 2-nitroimidazole molecule, from a crude extract of Streptomyces bacteria led to the identification of nitroimidazole antibiotics (20), and the chemical was subsequently found to have therapeutic potential against *Trichomonas vaginalis*, the protozoan responsible for the sexually transmitted infection trichomoniasis (21, 22).

Benznidazole, a member of the nitroimidazole family like 2-nitroimidazole, is widely recognized for its antiparasitic activity against *Trypanosoma cruzi*. It plays a key role in managing Chagas disease by not only reducing parasitic load and mortality but also minimizing inflammation and oxidative damage to tissues. Additionally, it offers immunomodulatory benefits, aiding in the regulation of inflammatory responses and providing protection to cardiac tissue (23, 24).

We hypothesized that 2-nitroimidazole could be effective in the treatment of toxoplasmosis. However, there is no information on its effect on the Toxoplasma parasite. This study is the first to evaluate the effect of 2-nitroimidazole on a virulent strain of *T. gondii* both in vitro and in vivo.

## 2. Objectives

Therefore, the aim of this study is to investigate the effects of 2-nitroimidazole on *T. gondii* using different tests such as tachyzoite assay, MTT assay, and flow cytometry.

## 3. Methods

This study received approval from the Ethical Committee of Tarbiat Modares University in Tehran, Iran (approval number: IR.MODARES.AEC.1402.028, dated September 30, 2023).

### 3.1. Drug Preparation and Toxoplasma gondii Parasite

The 2-nitroimidazole drug (molecular weight: 1113.07 daltons) was obtained from Sigma Company and prepared using a phosphate-buffered saline (PBS) solution (concentration: 40 μM; pH 7.4). The final concentration of the drug was adjusted to 40 μM in a total volume of 5 mL. Standard drugs for the treatment of toxoplasmosis (sulfadiazine) in dry powder form were purchased from Sobhan Daru Company. A sulfadiazine stock solution with a concentration of 1000 μg/mL was prepared by dissolving 10 mg of sulfadiazine powder in a mixture of 2 mL dimethyl sulfoxide (DMSO) and 8 mL distilled water. The solution was filtered through a 0.22-micron filter and stored in a freezer at -20°C. Subsequent concentrations of 40, 20, 10, 5, 2.5, 1.25, and 0.625 μM were prepared by diluting the stock solution in DMEM culture medium and stored at -4°C.

*Toxoplasma gondii* (RH strain) tachyzoites were obtained from the Department of Parasitology at the University of Tehran. They were diluted with PBS and maintained through serial passages in BALB/c mice. To preserve the parasite strain and prevent weakening, the tachyzoites were injected into the peritoneum of BALB/c mice for successive passages.

### 3.2. Macrophage Culture

The human monocytic leukemia cell line THP-1 was maintained in RPMI 1640 medium supplemented with 10% fetal bovine serum (FBS), 1% penicillin-streptomycin, and 1% L-glutamine. Cell proliferation was observed daily using an inverted microscope, and cell counting was performed using a 0.4% trypan blue solution. Upon reaching 80 - 90% confluence, the cells were trypsinized and subcultured into new flasks with fresh media. The cells were subsequently treated with 50 ng of phorbol myristate acetate (PMA) for 24 hours, followed by three washes. Afterward, the cells were allowed to rest for 24 hours.

### 3.3. Tachyzoite Assay

In this study, the toxic effect of 2-nitroimidazole on *T. gondii* was examined across seven different doses (40, 20, 10, 5, 2.5, 1.25, 0.625 μM) using 96-well plates. One well was used as a control (parasite without any drug). Tachyzoites were counted and morphologically assessed under light microscopy after exposure periods of three, six, and 24 hours, following staining with trypan blue dye. In addition to investigating the toxic effect of 2-nitroimidazole on *T. gondii*, the study also examined the impact of sulfadiazine at various concentrations (40, 20, 10, 5, 2.5, 1.25, 0.625 μM). This investigation was conducted using 96-well plates following the aforementioned protocol. The results obtained from both groups were subsequently compared to assess and contrast their respective toxic effects on *T. gondii*. It is important to note that all experiments were conducted in triplicate to ensure the reliability and consistency of the findings (25).

### 3.4. MTT Assay for Macrophages

To prepare the MTT solution, 5 mg of MTT powder from Sigma Chemical Co., Germany, was combined with 1 mL of sterile PBS in a dark room. Macrophage cells of the THP-1 cell line were cultured in RPMI 1640 medium supplemented with 10% FBS, 100 IU/mL penicillin, and 100 μg/mL streptomycin, and incubated at 37°C under 5% CO2. A 96-well culture plate was seeded with cells at a density of 5 × 10^5^ cells/mL, and 2-nitroimidazole was added at concentrations of 40, 20, 10, 5, 2.5, 1.25, and 0.625 μM. One well, serving as the control group, did not receive any 2-nitroimidazole treatment for the macrophage cells. After 72 hours of exposure, 20 μL of MTT solution was added to each well, and the plate was incubated at 37°C for 5 hours to allow the cells to convert the tetrazolium salt into insoluble formazan crystals. The supernatant was carefully removed from the wells, and 100 μL of DMSO was added to each well in a dark place. The optical density (OD) was measured at 540 nm using an ELISA reader device (Stat Fax, USA). Finally, the percentage of cell viability in both exposed and control groups was calculated using the following formula (26).

Viable macrophages (%) = (AT-AB)/ (AC-AB) × 100

AT: Absorbance of exposed macrophages

AC: Absorbance of unexposed macrophages

AB: Absorbance of the blank

### 3.5. Flow Cytometry

This research utilized flow cytometry with dual staining, incorporating annexin V (V-FLUOS) and propidium iodide (PI), to evaluate the type of cell death, distinguishing between apoptosis and necrosis. In this methodology, the device output is divided into four quadrants, each representing different cell states: The lower right quadrant, stained with annexin V, indicates apoptotic cells; the upper right quadrant, stained with both annexin V and PI, signifies delayed or secondary apoptosis; the lower left quadrant, which remains unstained, corresponds to living cells; and the upper left quadrant, stained with PI, denotes necrotic cells.

In our study, we used the Annexin V-FITC apoptosis kit (BioVision) for flow cytometry. Following the kit instructions, we treated 2 × 10^5^ tachyzoites with 5.43 μM (IC50 concentration) 2-nitroimidazole and 2.99 μM (IC50 concentration) sulfadiazine. After 24 hours of incubation at 24°C, 500 µL of buffer was added to the samples, which were then placed on ice for 15 minutes. Subsequently, 5 µL of PI and annexin V were added, and the samples were analyzed using a flow cytometry device. The device presented the results in the form of a graph. Data were analyzed using FlowJo software and expressed as percentages.

### 3.6. Experimental Animal and in vivo Assay

Twenty female BALB/c mice, weighing between 20 and 25 grams, were purchased from the Razi Vaccine and Serum Research Institute in Karaj, Iran. These mice were divided into four groups, each containing five mice, as follows:

(1) Control group: Received no medication.

(2) Standard sulfadiazine group: Received 100 microliters of the standard sulfadiazine drug (5 doses administered every other day).

(3) 2-nitroimidazole (12 micrograms) group: Received 100 microliters of the 2-nitroimidazole drug containing 12 micrograms (5 doses administered every other day).

(4) 2-nitroimidazole (24 micrograms) group: Received 200 microliters of the 2-nitroimidazole drug containing 24 micrograms (5 doses administered every other day).

Before conducting experiments and administering drugs, 10^4^ fresh *T. gondii* tachyzoites were injected intraperitoneally into BALB/c mice to induce infection. The injections were administered five times, with one-day intervals between each injection. All groups were monitored and examined until the completion of the 14th day.

### 3.7. Statistical Analysis

Statistical analyses were conducted using IBM SPSS Statistics Version 21, with data visualization carried out using GraphPad Prism Version 8.0.1 and Microsoft Office Excel 2013. Parametric tests, including the unpaired samples *t*-test, were employed to compare results between the treatment and control groups. Differences were considered significant at a 5% level (P < 0.05) compared to the control.

## 4. Results

### 4.1. Tachyzoite Assay

**Figure 1.**
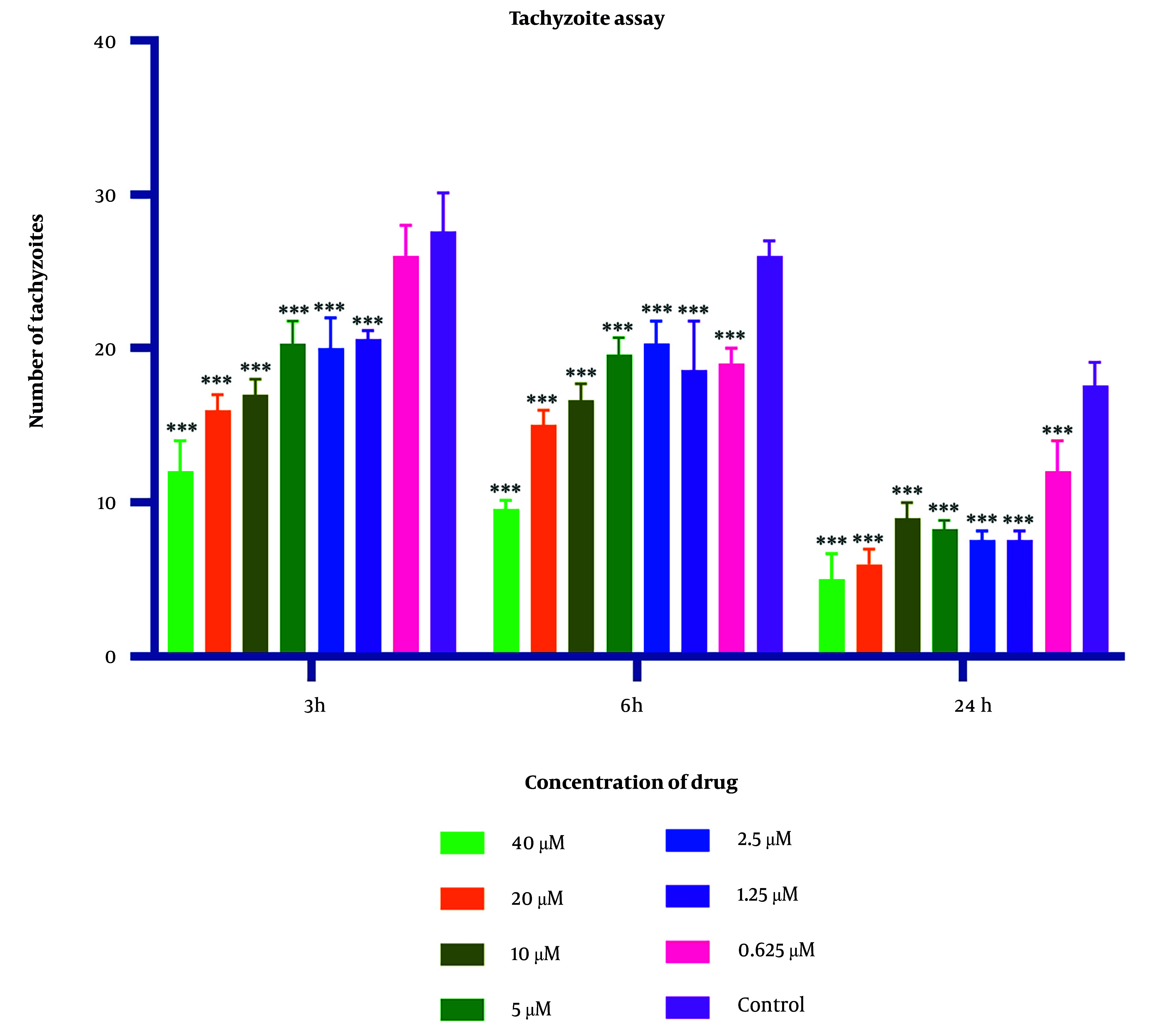
Mean and standard deviation of *Toxoplasma gondii* tachyzoite quantities demonstrated variability across diverse 2-nitroimidazole concentrations over periods of 3, 6, and 24 hours, in contrast to the untreated control cohort. Statistical analysis revealed significant disparities between the experimental groups exposed to the compound and the unexposed control group (statistical significance: P < 0.05 relative to control,*** P < 0.001. Each experiment is performed in triplicate).

**Figure 2.**
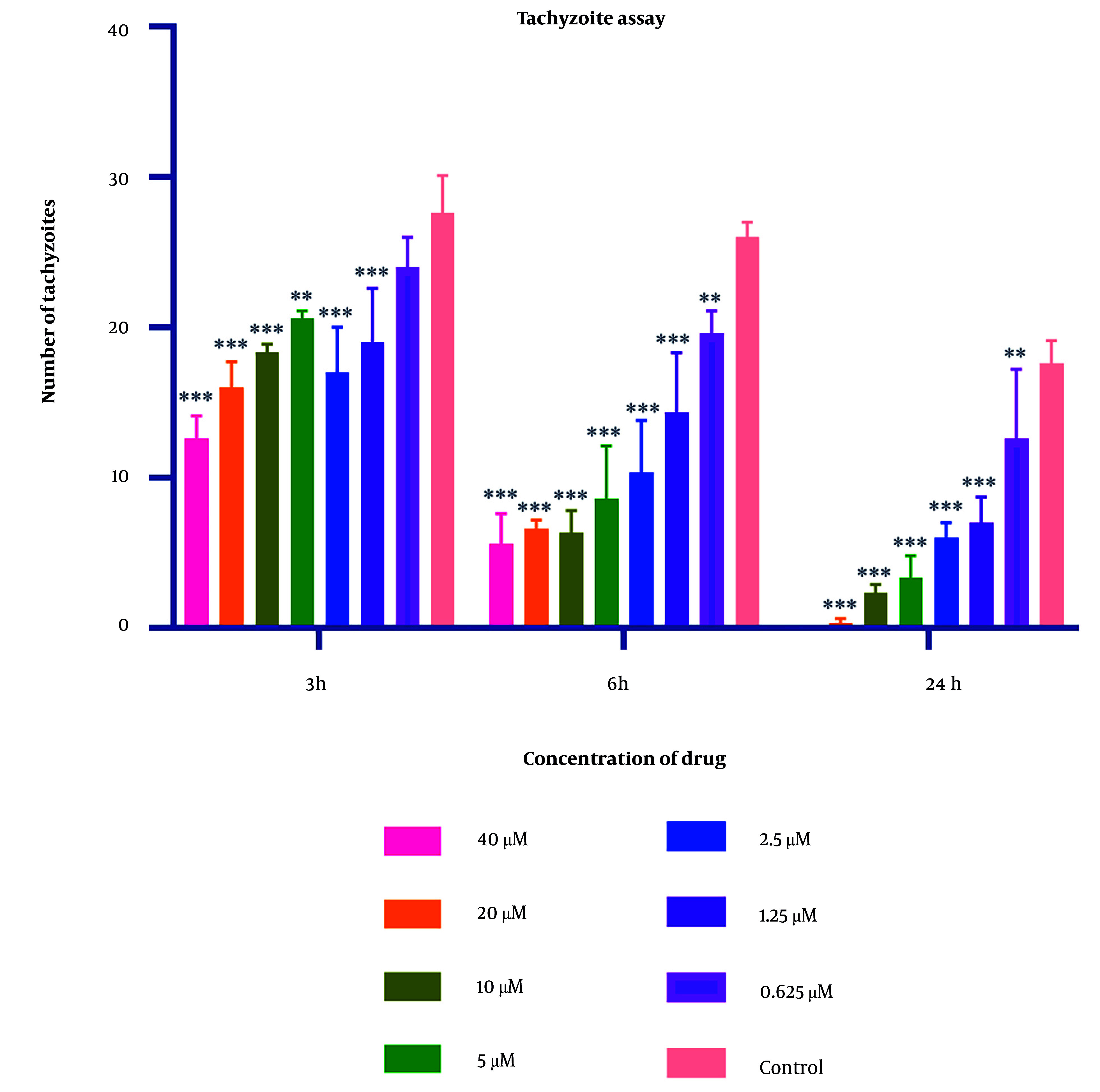
Mean and standard deviation of *Toxoplasma gondii* tachyzoite quantities demonstrated variability across diverse sulfadiazine concentrations over periods of 3, 6, and 24 hours, in contrast to the untreated control cohort. Statistical analysis revealed significant disparities between the experimental groups exposed to the compound and the unexposed control group (statistical significance: P < 0.05 relative to control,** P < 0.01, *** P < 0.001. Each experiment is performed in triplicate).

Parasite counts subjected to varying concentrations of 2-nitroimidazole and sulfadiazine were assessed using optical microscopy at intervals of 3, 6, and 24 hours. The IC50 values for 2-nitroimidazole and sulfadiazine, as determined through microscopic observation, were found to be 5.43 μM and 2.99 μM, respectively. The findings indicated a positive correlation between the concentration of 2-nitroimidazole and sulfadiazine and their lethality towards the parasite, with higher concentrations resulting in increased mortality. Additionally, prolonged exposure to 2-nitroimidazole and sulfadiazine led to a greater reduction in tachyzoite counts. At all concentrations except for 0.625 μM, the differences compared to the control group were statistically significant (P < 0.05). Light microscopy revealed notable morphological changes in parasites exposed to these drugs. Detailed data are presented in Figures 1 and 2. 

### 4.2. MTT Assay for Macrophage

**Figure 3.**
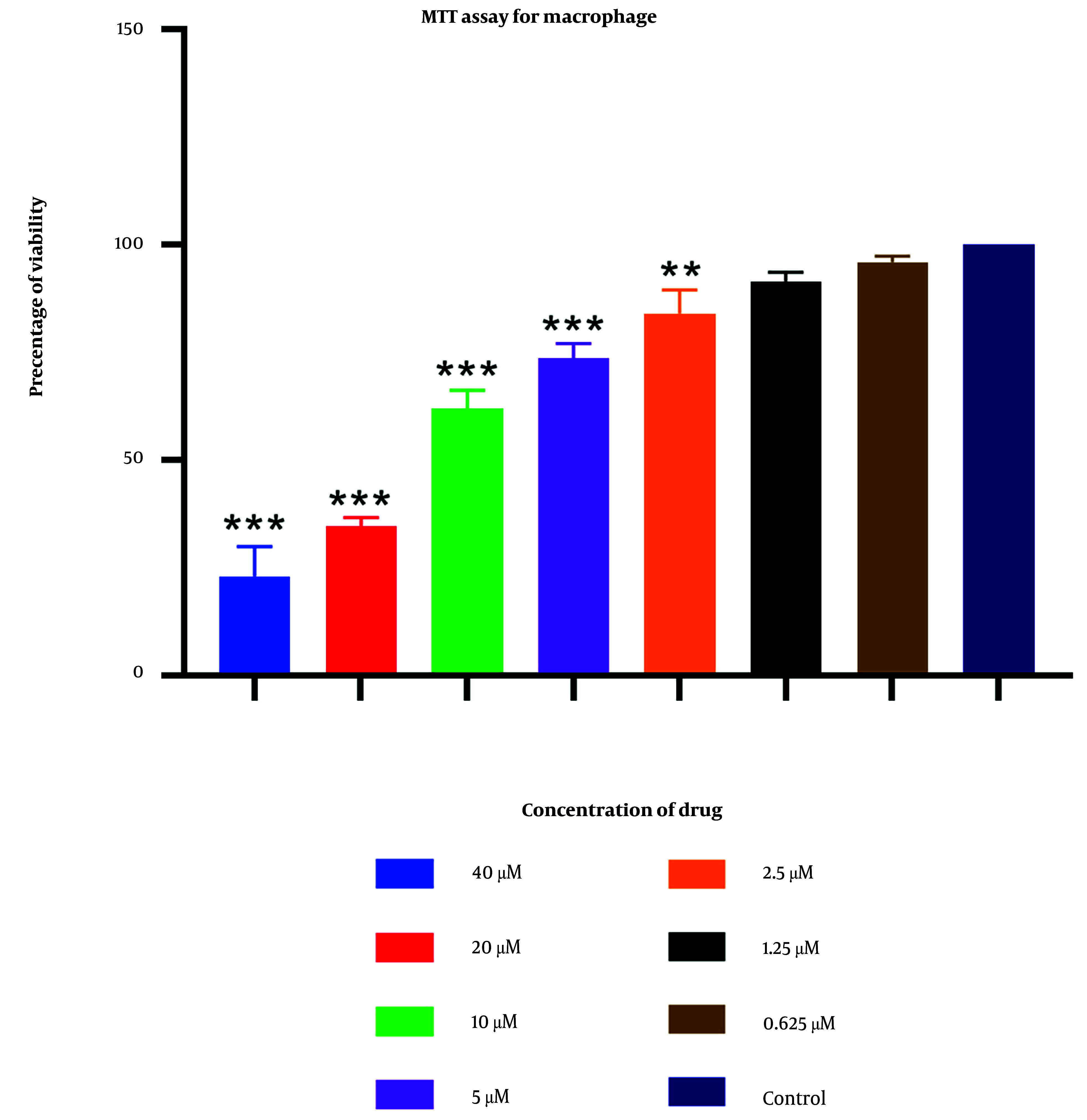
Mean and standard deviation of macrophage viability percentages following 24-hour exposure to varying 2-nitroimidazole concentrations. (statistical significance: P < 0.05 relative to control, ** P < 0.01, *** P < 0.001. Each experiment is performed in triplicate).

An MTT assay was utilized to determine the cytotoxic concentrations of 2-nitroimidazole on macrophage cells. The results indicated that at the highest concentration tested (40 μM), the maximum observed lethality was 77% (Figure 3). In contrast, other concentrations of extracts exhibited negligible effects, showing no significant differences compared to the control group. At a concentration of 0.625 μM, over 95% of macrophages remained viable. Further details are provided in Figure 3. 

### 4.3. Flow Cytometry Analysis

**Figure 4.**
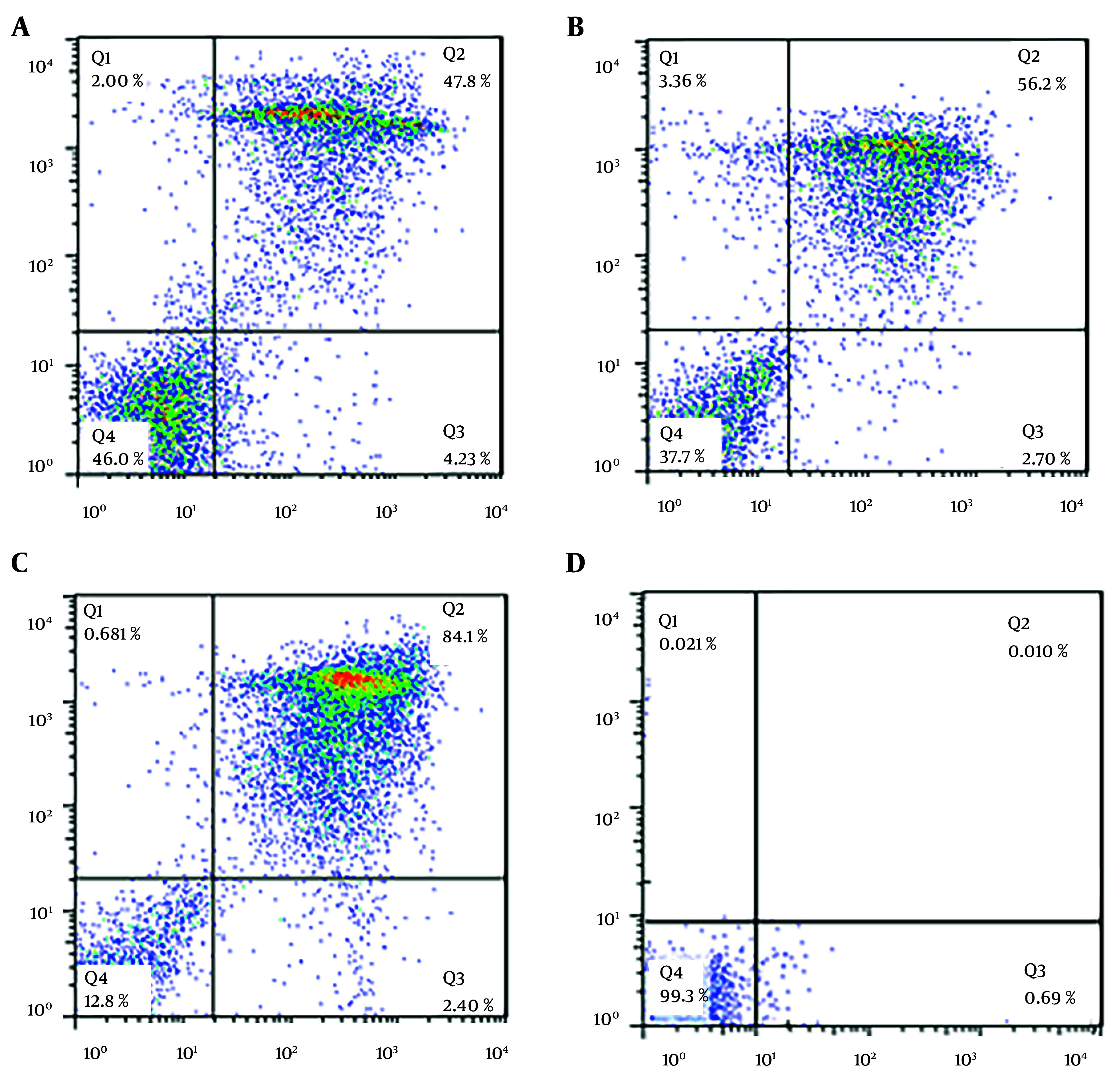
Flow cytometry analysis of tachyzoite viability under four groups: A, 10 μM 2-nitroimidazole treatment; B, 20 μM 2-nitroimidazole treatment; C, 100 mM sulfadiazine treatment; D, control group

A concentration-dependent induction of apoptosis was observed in tachyzoites following 24 hours of treatment with 2-nitroimidazole and sulfadiazine. In this study, a flow cytometry assay was utilized to assess the percentages of necrotic, apoptotic (both primary and secondary), and live cells. For tachyzoites treated with 2-nitroimidazole at a concentration of 100 μg/mL, the percentages of apoptotic, necrotic, and live cells were 52%, 2%, and 46%, respectively (Figure 4A). At a concentration of 200 μg/mL, treatment with 2-nitroimidazole resulted in 58.9% apoptotic, 3.36% necrotic, and 37.7% live cells (Figure 4B). Similarly, for tachyzoites treated with sulfadiazine at a concentration of 100 μg/mL, the percentages of apoptotic, necrotic, and live cells were 86.5%, 0.68%, and 12.8%, respectively (Figure 4C). In the control group, the percentages of live, necrotic, and apoptotic cells were 99.3%, 0.02%, and 0.70%, respectively. The flow cytometry results are presented in Figure 4D. 

### 4.4. In vivo Assay

**Figure 5.**
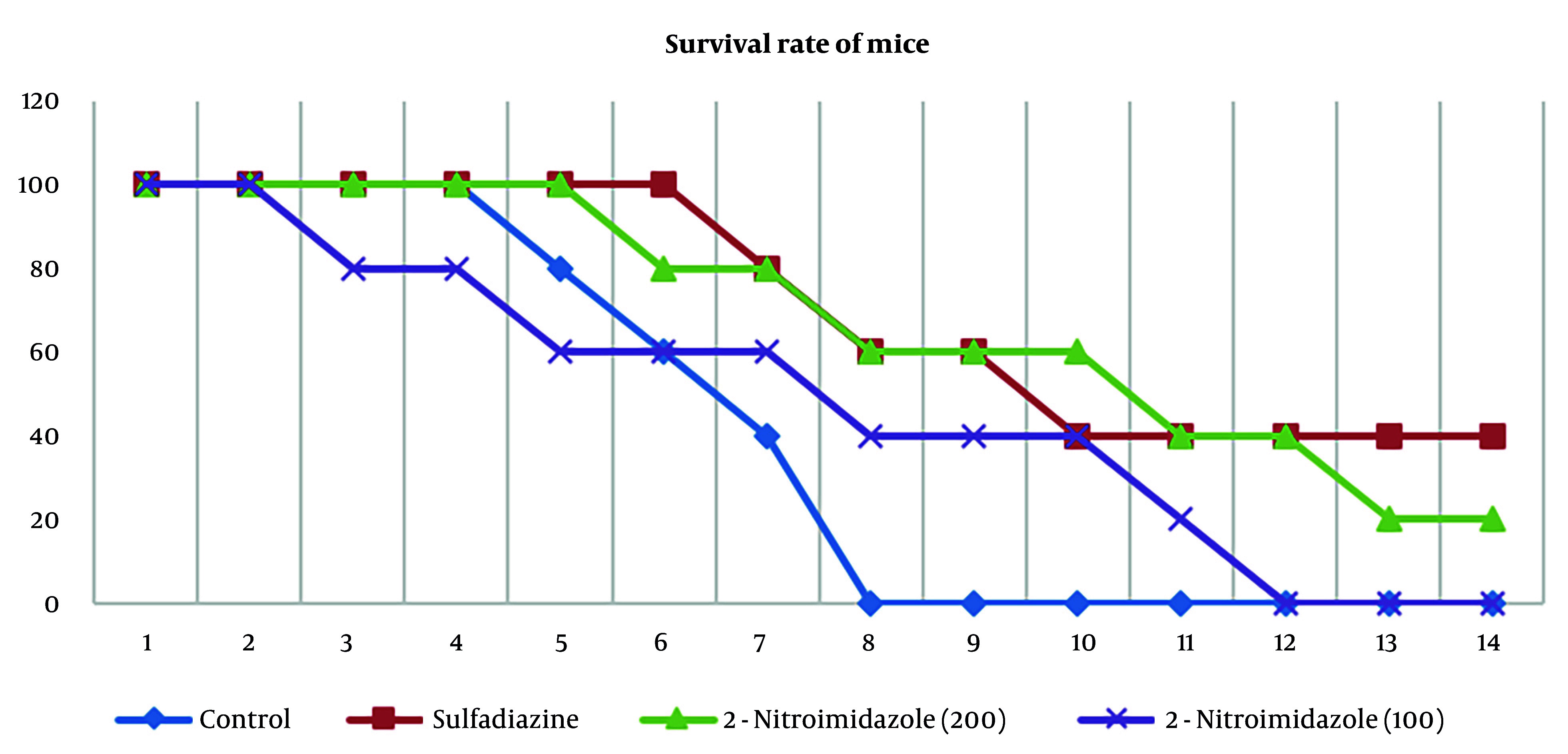
Survival rates of *Toxoplasma gondii*-infected BALB/c mice over a 14-day post-challenge period, comparing untreated controls with groups receiving: 2-nitroimidazole at 100 μg/μL, 2-nitroimidazole at 200 μg/μL and sulfadiazine at 100 μg/μL (n = 5 per group)

The administration of 2-nitroimidazole and sulfadiazine significantly increased the lifespan of treated mice compared to the control group. All groups were monitored until the end of the 14th day. According to the results, all mice in the control group died by the eighth day. Sulfadiazine treatment yielded the best outcome, with two mice surviving until the 14th day, followed by 2-nitroimidazole treatment, where one mouse survived until the 14th day. The survival percentages across different groups are depicted in Figure 5. 

## 5. Discussion

Insufficient hygiene practices are a significant factor in the spread of toxoplasmosis. Studies have shown that around 30% of the global population is infected with *T. gondii* (4). Toxoplasmosis poses a significant threat to immunocompromised individuals, including those with malignancies, HIV/AIDS patients, and organ transplant recipients, with symptoms encompassing encephalitis, seizures, and vision disorders (5, 6). There is an urgent necessity to develop novel, safe, effective, and well-tolerated pharmacological treatments for toxoplasmosis (27). Current anti-*T. gondii* therapies are unable to eliminate parasitic cysts, and patients with compromised immune systems often cannot tolerate these medications (8). Pyrimethamine and sulfadiazine are first-line medications for treating and preventing toxoplasmosis, but they can cause adverse effects like neutropenia, leukopenia, thrombocytopenia, blood abnormalities, allergic reactions, and increased serum enzymes (7). Given that severe and life-threatening complications of toxoplasmosis remain unresolved on a global scale, prioritizing the development of novel anti-toxoplasma therapies is imperative to combat the infection and mitigate its spread (28, 29).

To date, research has focused on the antibacterial and antiparasitic properties of 2-nitroimidazole, including its effects on *Giardia* *lamblia* and *T.* *cruzi*. However, there have been no published studies investigating the anti-toxoplasmic activity of 2-nitroimidazole (30-32). This study represents the initial investigation into the effects of 2-nitroimidazole on *T. gondii* infections and its comparison with sulfadiazine, both in vitro and in vivo.

In this investigation, varying concentrations of 2-nitroimidazole and sulfadiazine were administered in culture plates containing *T. gondii*. According to the counting of tachyzoites with a hemocytometer slide and light microscope, the data revealed that higher concentrations of these drugs, combined with extended exposure durations to tachyzoites, substantially increased the lethal effect on the parasite. Significantly, the highest mortality rate of tachyzoites was observed with 2-nitroimidazole at a concentration of 40 μM and with sulfadiazine at a concentration of 40 μM following a 24-hour exposure period.

The results of this study suggest that 2-nitroimidazole holds promise as a potential therapeutic agent for *T. gondii*, demonstrating efficacy comparable to the standard treatment, sulfadiazine, with potentially fewer side effects. In vitro, 2-nitroimidazole exhibited a potent anti-parasitic effect, with an IC50 value of 5.43 μM, which was slightly higher than that of sulfadiazine (2.99 μM). In agreement with our study, a 2022 study by Faria et al. synthesized two novel 2-nitroimidazole compounds and evaluated their efficacy in L929 cell cultures infected with *T. cruzi*. The most promising compound, the 5b 2-nitroimidazole derivative, demonstrated significant anti-trypanosomal activity, with a half-maximal inhibitory concentration (IC50) of 2.3 μM (33). These findings highlight the anti-toxoplasmic activity of 2-nitroimidazole, emphasizing its potential as an effective treatment for toxoplasmosis.

Additionally, the MTT assay was employed to assess the potential cytotoxicity of 2-nitroimidazole on macrophages. The results indicated a high survival rate of macrophages at lower concentrations of 2-nitroimidazole, demonstrating minimal toxicity. Specifically, at concentrations of 2.5 μM, 1.25 μM, and 0.625 μM, the survival rates of macrophages were 84%, 91.5%, and 96%, respectively. This aspect is vital, as an optimal therapeutic agent must effectively target the parasite while preserving the viability of host cells. The combination of low cytotoxicity and potent anti-tachyzoite activity positions 2-nitroimidazole as a promising candidate for further development and potential clinical application in toxoplasmosis treatment. This is a notable advantage over sulfadiazine, which is associated with a range of adverse effects, including neutropenia, leukopenia, and thrombocytopenia.

Additionally, these findings could encourage further research to optimize dosing regimens and explore the synergistic potential of these drugs in combination therapy. Flow cytometry analysis revealed that 2-nitroimidazole and sulfadiazine induced apoptosis in approximately 58.9% and 86.5% of tachyzoites, respectively. The flow cytometry analysis revealed that 2-nitroimidazole induced apoptosis in a significant proportion of *T. gondii* tachyzoites (58.9%) with minimal necrosis, supporting its targeted action against the parasite without excessive host cell damage. This is particularly important as effective anti-parasitic treatments should minimize harm to the host, especially in immunocompromised individuals who are more vulnerable to treatment-related side effects.

Both MTT and flow cytometry analyses validated the efficacy of 2-nitroimidazole as a viable therapeutic option for toxoplasmosis. To further substantiate these findings, 2-nitroimidazole and sulfadiazine were administered to BALB/c mice for treatment evaluation. All mice in the control group perished by the eighth day. In vivo, while sulfadiazine provided the best survival outcome, with two mice surviving until day 14, 2-nitroimidazole demonstrated comparable efficacy, with one mouse surviving to the same endpoint. These findings highlight the potential of 2-nitroimidazole as an effective alternative to sulfadiazine, with the added benefit of lower cytotoxicity to host cells.

Given these findings, 2-nitroimidazole presents as a promising candidate for further development, particularly in combination therapies or as an adjunct to standard treatments. Further research, including optimizing dosing regimens and exploring its efficacy in chronic and latent stages of *T. gondii* infection, will be essential in determining its clinical applicability. Additionally, the lower cytotoxicity observed in this study suggests that 2-nitroimidazole may offer a safer alternative, particularly for immunocompromised patients who require long-term treatment.

### 5.1. Conclusions

This research underscores the potential of 2-nitroimidazole as a treatment option for *T. gondii*. The findings from both in vitro and in vivo experiments revealed notable anti-tachyzoite effects and low toxicity, suggesting it could be a valuable candidate for further study. While sulfadiazine continues to be the standard therapy, 2-nitroimidazole demonstrated similar effectiveness and may present a safer alternative with fewer adverse effects. To fully realize its clinical value, future work should focus on larger sample sizes, expanded dosing studies, and combination approaches to refine its therapeutic use.

## Data Availability

The dataset presented in the study is available on request from the corresponding author during submission or after publication.
